# Competency-based curricula in the Eastern Mediterranean Region schools of pharmacy: a framework-informed mixed method study

**DOI:** 10.1080/20523211.2026.2612865

**Published:** 2026-01-21

**Authors:** Muna Al-Ismail, Reem El-Hage, Ahmed Awaisu, Somaya Mahmoud, Mariam Mustafa, Mohammed Al-Hamdani, Banan Mukhalalati

**Affiliations:** a Department of Clinical Pharmacy and Practice, College of Pharmacy, Health Sector, Qatar University, Doha, Qatar; b Department of Public Health, College of Health Sciences, Health Sector, Qatar University, Doha, Qatar; c Health Sector, Qatar University, Doha, Qatar

**Keywords:** Pharmacy education, competency-based education, accreditation, framework, Eastern Mediterranean Region

## Abstract

**Background:**

Competency-based education (CBE) has been implemented across healthcare disciplines, including pharmacy, to address the limitations of the traditional teaching system. While CBE implementation in pharmacy education has been studied globally, research examining its adoption across pharmacy schools in the Eastern Mediterranean Region (EMR) remains limited.

**Aim:**

Explore the nature and extent of CBE incorporation within pharmacy schools' curricula across the EMR and identify barriers and facilitators influencing CBE implementation.

**Methods:**

A sequential explanatory mixed-methods approach was used, combining an online survey followed by semi-structured online interviews. The study was guided by the International Pharmaceutical Federation Global Competency Framework (FIP GbCF).

**Results:**

Out of the 116 schools that received the survey link, 55 responses were received from pharmacy schools in the EMR (any-response rate 47.4%), of which 38 were complete and included in the final analysis (complete-response rate 32.8%). Furthermore, 18 participants participated in the online interview. The majority of the pharmacy schools were accredited (81.6%), and (63.2%) of these implement/incorporate CBE in their curricula. The analysis of the FIP GbCF competencies incorporation revealed that various competencies were prevalent, including communication skills and professional and ethical practice (95.8%), and dispensing and inter-professional collaboration (91.7%). The least incorporated competencies included emergency response (58.3%), followed by workplace management (25%). Inductive thematic analysis identified key challenges for the implementation of CBE in some pharmacy schools, including internal resistance, resource limitations and workload challenges. On the other hand, schools highlighted important facilitators such as leadership support, communication and engagement.

**Conclusion:**

Most accredited pharmacy schools in the EMR adopted CBE, yet considerable gaps remain in implementing many of the FIP GbCF competencies. The study reveals that pharmacy schools face significant challenges in CBE implementation, regardless of their current adoption status, suggesting opportunities for the enhancement of pharmacy education across the region.

## Background

The concept of competency-based education (CBE), defined as the integration of knowledge with learners’ skills and abilities to facilitate the application of knowledge in real-world practice, emerged in the 1960s (Bajis et al., 2016) and continues to evolve. CBE was created to combat traditional educational systems that fail to expose students to critical thinking and decision-making in clinical contexts (Shah et al., 2016). With technological advancements, an ageing population, and chronic disease burdens, an educational reform became necessary (Nilsen et al., 2020). In response, CBE has been adopted across various healthcare disciplines, including pharmacy, to develop skilled professionals who can meet these evolving demands (Nilsen et al., 2020; Shah et al., 2016). The application of CBE in pharmacy, known as competency-based pharmacy education (CBPE), plays an essential role in training pharmacists to become competent healthcare professionals (Bruno et al., 2010; Nash et al., 2015). The benefits of applying CBPE to achieve established competency standards include identifying graduate competencies, making learning individualised, and ensuring similar baseline skills across programmes and regions (Frank et al., 2010; Medina, 2017; Shah et al., 2016; ten Cate, 2005). Recent studies have continued to explore the use of CBE in pharmacy and other health professions. A systematic review by McMullen et al. showed that CBE approaches are being increasingly used in pharmacy programmes around the world, focusing on practical learning and assessment of real skills. However, the review also noted that the level of implementation differs between countries and institutions (McMullen et al., 2023). Another recent study reported that although CBE helps improve learners’ competence and preparation for clinical practice, challenges such as limited faculty training, complex assessment processes, and institutional barriers remain (Jarrett et al., 2024). These studies highlight the growing global interest in CBE and the need to understand how such approaches are being applied in different regional and resource contexts, including in the EMR.

A key factor in achieving the required competencies in pharmacy education is the use of competency frameworks, which define the essential skills and behaviours for practice performance (Brown et al., 2012) and guide CBE implementation (Atkinson et al., 2015). Many frameworks exist globally (Bajis et al., 2020; Croft et al., 2019). However, there is no universally accepted gold standard competency framework to follow, as frameworks must align with local societal needs (Koster et al., 2017). The first globally applicable framework, known as the International Pharmaceutical Federation Global Competency Framework (FIP GbCF), was developed in 2012 by the Pharmacy Education Taskforce, a collaboration among FIP, World Health Organization (WHO), and the United Nations Educational, Scientific and Cultural Organization (UNESCO) (FIP Academic Pharmacy Section, 2022; International Pharmaceutical Federation, 2023; Katoue & Schwinghammer, 2020). The FIP GbCF includes four main domains, serving as a tool for mapping and advancing pharmacy education and practice in different regions globally: (1) pharmaceutical public health, (2) pharmaceutical care, (3) organisation and management, and (4) professional/personal competencies (FIP Academic Pharmacy Section, 2022; International Pharmaceutical Federation, 2023). Other examples of competency frameworks include the Center for the Advancement of Pharmacy Education (CAPE) Educational Outcomes by the American Association of Colleges of Pharmacy (AACP) (American Association of Colleges of Pharmacy, 2013) and the Professional Competencies for Canadian Pharmacists at Entry to Practice by the National Association of Pharmacy Regulatory Authorities (NAPRA) (The National Association of Pharmacy Regulatory Authorities, 2024). However, these frameworks are designed for local use (Austin & Ensom, 2008; Medina, 2017), although they are adopted by several other countries.

While CBPE is well established in Western countries and more recently adopted in East Asia, its implementation remains underdeveloped in many developing countries, including those in the Eastern Mediterranean Region (EMR) (Fittler et al., 2022; Jacob et al., 2021; Katoue & Schwinghammer, 2020; Maitreemit et al., 2008; Meilianti et al., 2021; Mucalo et al., 2016; Walter et al., 2018). The limited adoption of CBPE in the EMR may be due to challenges such as the need for strong institutional leadership, comprehensive curriculum design, implementation strategies, ongoing faculty development, and a lack of workforce (Hajj et al., 2023; Katoue & Schwinghammer, 2020). A regional study in 15 EMR countries assessing the perspective of pharmacy stakeholders on pharmacy education reported that the absence of local frameworks and variability in pharmacy education hinder CBE implementation (Bajis et al., 2018). This was further emphasized by a study conducted in Lebanon, noting that international frameworks may not suit resource-limited contexts, prompting the development of a national framework (Sacre et al., 2022).

A comprehensive literature review revealed that no previous study has assessed the application of CBE in pharmacy across all 22 EMR countries (Abrika et al., 2012; Al-Ghamdi, 2001; Bajis et al., 2016, 2018; Hasan, 2009; Katoue & Schwinghammer, 2020; Sacre et al., 2022; Wilbur, 2010). Available studies mostly explored the perceptions of students or pharmacists or described educational experiences related to competencies (Abrika et al., 2012; Al-Ghamdi, 2001; Hasan, 2009; Kheir et al., 2017; Mukhalalati et al., 2021; Wilbur, 2010). A prior review on CBPE in 13 EMR countries also suggested the need for more research on the barriers and facilitators influencing CBPE implementation in the region (Bajis et al., 2016).

Therefore, this study represents the first systematic exploration of the nature and extent of CBE incorporation in pharmacy schools’ curricula across all EMR countries. The objectives of the study were to: (1) assess the current status of CBE implementation in EMR pharmacy schools’ curricula and (2) identify the key barriers and facilitators influencing the adoption of CBE. The findings of this study are intended to guide institutional leaders and policymakers by providing evidence to support the enhancement of pharmacy education and curriculum development in the region.

## Methods

### Study design

This study utilised a sequential explanatory mixed-methods design, with greater analytical emphasis placed on the qualitative component, to comprehensively explore the implementation of CBE across pharmacy schools in the EMR. The study placed greater analytical emphasis on the qualitative component, and the quantitative findings were intended to provide contextual and descriptive support rather than inferential conclusions (Ivankova et al., 2006). The quantitative phase involved a cross-sectional study using a web-based, self-administered questionnaire conducted between March 2022 and August 2023. This was followed by a qualitative phase employing a phenomenological approach, using semi-structured online interviews conducted between October and December 2024 to further explore and contextualise the survey findings.

The qualitative component was guided by a descriptive interpretivist approach to explore participants’ lived experiences and perspectives in depth regarding CBE implementation. The study included all pharmacy schools within the EMR and was reported in accordance with the Strengthening the Reporting of Observational Studies in Epidemiology (STROBE) guidelines and the Standards for Reporting Qualitative Research (SRQR) guidelines (O'Brien et al., 2014; Von Elm et al., 2007).

### Study participants

The study targeted pharmacy schools offering Bachelor of Pharmacy (BSc) and Doctor of Pharmacy (PharmD) degree programmes within the EMR, regardless of their language of instruction or accreditation status. Countries in the EMR (as defined by the WHO at the time of study planning (World Health Organization, 2020)) were 22 and included: Afghanistan, Bahrain, Djibouti, Egypt, Iran, Iraq, Jordan, Kuwait, Lebanon, Libya, Morocco, Oman, Pakistan, Palestine, Qatar, Saudi Arabia, Somalia, Sudan, Syria, Tunisia, the United Arab Emirates, and Yemen. Pharmacy schools offering only postgraduate (MSc or PhD) degrees were excluded due to differing competency requirements and educational objectives (Frank et al., 2010).

### Participant identification and recruitment

Pharmacy schools were identified using the FIP World List of Pharmacy Schools (International Pharmaceutical Federation, 2022). Contact details for relevant academic administrators (e.g. Deans, Vice Deans, or Curriculum Committee Chairs) were obtained via official institutional websites and, when unavailable, through university administrative offices and professional social media platforms. All eligible schools were contacted via email and invited to participate in the study, with the option to nominate a suitable delegate if the initial contact was unable to participate. All individuals who consented to participate were sent an email containing the online survey link. Similar procedures were applied for recruitment to the qualitative interview phase, with email invitations sent one to two weeks in advance, along with a participant information leaflet and informed consent form. For the qualitative phase, a purposive and pragmatic sampling strategy was used to ensure inclusion of pharmacy schools with varied experiences, including those implementing and not implementing CBE, in alignment with the study objectives.

### Sample size

Using the Raosoft® online sample size calculator (Raosoft, 2004), a minimum sample size of 111 schools was determined based on a total population of 154 pharmacy schools in the EMR, a 95% confidence level, and a 5% margin of error. Given the relatively small population size, a census approach was adopted in accordance with recommended practice for small populations (Fink, 2013), and all of the 154 potentially eligible pharmacy schools were contacted. The final accessible population was 131 schools, since 23 institutions had no valid contact information. For the qualitative phase, a purposive sampling technique was employed to invite a target of approximately 20 schools (both implementing and not implementing CBE) for interviews.

### Data collection tools

#### Quantitative phase – self-administered online survey

Since there were no available survey instruments in the literature to address the specific objectives of this study, the research team developed survey items based on an extensive review of the literature (Abrika et al., 2012; Al-Ghamdi, 2001; Bajis et al., 2016, 2018; FIP Academic Pharmacy Section, 2022; Hasan, 2009; International Pharmaceutical Federation, 2025; Katoue & Schwinghammer, 2020; Sacre et al., 2022; Wilbur, 2010) and expert opinion meetings. The survey development was also guided by the FIP GbCF as it was the only global competency framework that could be applied to different countries (FIP Academic Pharmacy Section, 2022; International Pharmaceutical Federation, 2023). In addition, the FIP’s Global Framework for Quality Assurance of Pharmacy Education was extensively utilised to inform survey content development (Rouse & Meštrovic, 2014).

Content and face validity were established through review by three experts (two academic faculty and one FIP-affiliated expert). The instrument underwent four revisions to incorporate the experts’ feedback, including adoption of a semantic differential scale and integration of the Consolidated Framework for Implementation Research (CFIR) for comprehensiveness (Damschroder et al., 2022). A pilot test was conducted with three experienced faculty members to assess clarity, readability, and time burden.

The final questionnaire consisted of five main sections: [1] introduction and definitions; [2] informed consent; [3] background information (14 items); [4] development and implementation of CBE (8 items with 23 sub-items, mapped across the four FIP GbCF domains); and [5] perceived facilitators (12 items) and barriers (7 items) to CBE implementation. Items were rated on semantic differential scales (0 = not at all incorporated/perceived; 5 = highly incorporated/perceived). Open-text fields were included at the end of each section for additional comments.

The survey was administered online via SurveyMonkey® (SurveyMonkey Inc., San Mateo, CA, USA). It was initially opened from March 25, 2022, until December 1, 2022, and was later extended to August 2023 due to low response rates. Several reminder emails were sent at biweekly intervals, followed by telephone outreach for non-respondents. Final data were exported and analysed using IBM SPSS® Statistics for Windows, version 29.0 (IBM Corp., Armonk, NY, USA). Schools that had not yet implemented any form of CBE were instructed not to complete the survey as the questionnaire items required direct experience with CBE implementation. This approach was adopted to ensure data validity. However, these schools were invited to participate in the qualitative phase, where their perspectives on perceived barriers and potential facilitators for future CBE implementation were explored in depth.

#### Qualitative phase – semi-structured online interviews

The interview guide focused on exploring perceived facilitators and barriers to implementing CBE. The semi-structured interview guide was developed by the research team based on the study objectives and findings from the quantitative phase, and it was used flexibly to allow participants to elaborate on issues they perceived as most relevant. As the study target population comprised the pharmacy schools in the EMR, interviews were conducted online via video conferencing (Microsoft Teams or other preferred platforms) (Sah et al., 2020; Sedgwick & Spiers, 2009). Eighteen online interviews were conducted by a team of two trained moderators: one led the interviews, while the other assisted in the facilitation, note-taking, monitoring time, and ensuring the smooth operation of technical equipment. Each interview lasted approximately one hour and was conducted in English. All sessions were audio-recorded with participant consent and were transcribed verbatim (Elizabeth & Patricia, 2006).

The transcripts were subsequently and independently reviewed for content and accuracy by two researchers (MA and RA). All identifying information in the transcripts was anonymized using alphanumeric codes: ‘CBE' for pharmacy schools that implemented CBE, ‘non-CBE' for pharmacy schools that did not implement CBE. Inductive thematic analysis was employed to analyse the interview data. The interviews were conducted by researchers with training and experience in qualitative research. Reflexivity was addressed through ongoing awareness of researchers’ potential assumptions and regular team discussions during data collection and analysis to minimise interpretive bias.

### Ethical consideration

This study was granted ethical approval from the Qatar University Institutional Review Board (QU IRB) (approval number QU-IRB 1637-EA/21). Participation in the study was entirely voluntary, and informed consent was obtained from all participants prior to data collection. Confidentiality and anonymity were maintained throughout the study, and participants were informed of their right to withdraw at any stage without any consequences.

### Data analyses

Survey data were analysed using descriptive statistics. Frequencies and percentages were used to report demographic characteristics, levels of FIP GbCF domains implementation, and perceived facilitators and barriers to CBE implementation. Schools that had not implemented any form of CBE were not required to complete Sections 4 and 5 on the FIP GbCF and were therefore excluded from the quantitative analysis of these sections. For the qualitative component, data were analysed thematically following an iterative process of familiarisation, coding, and theme development (Braun & Clarke, 2006). Two researchers (MA and RA) independently coded the interview transcripts using inductive thematic analysis. A third researcher (MH) reviewed all the coding and emerging themes to ensure consistency and accuracy. In cases of discrepancies during data coding and interpretation, consensus was sought between the two researchers; if unresolved, a third researcher (AA) was consulted to reach a final decision. As shown above, methodological rigour was enhanced through investigator triangulation, iterative review of emerging codes and themes, and consensus-based resolution of discrepancies.

To provide a comprehensive understanding of the facilitators and barriers towards implementing CBE, the qualitative and quantitative data were integrated at the interpretation and reporting stage. A narrative, contiguous approach was used, whereby the findings of the two phases were included in a single report, under separate sections (Fetters et al., 2013). Themes derived from both datasets were compared to identify similarities and differences, and the results were aligned under corresponding thematic categories (Fetters et al., 2013). However, greater interpretive emphasis is placed on the qualitative findings, which provide richer contextual understanding of the phenomena under investigation.

## Results

### Quantitative phase

#### Characteristics of pharmacy schools in the EMR

In the quantitative phase, the survey was sent to 131 pharmacy schools across the EMR, but emails were undelivered to 15 schools. Of the 116 schools that received the survey, 55 responded (any-response rate: 47.4%). Out of these responses, one declined participation, and 16 surveys were incomplete (omitting sections 4 and 5), resulting in 38 complete responses (complete-response rate: 32.8%) from institutions in 14 different countries. The demographic characteristics of the schools that participated in this phase are presented in Table 1. Half were public institutions, and 58% offered both BSc and MSc degree programmes. The number of admitted students into different programmes was less than 150 in the majority of the schools. The accreditation status and competency framework adaptation/adoption for the participating schools are presented in Table 2. Among the 38 responding schools, the majority were accredited (n = 31; 81.6%) and implemented/incorporated CBE in their curricula (n = 24; 63.2%). The majority of the schools implementing CBE reported the utilisation of at least one competency framework, including the AACP-CAPE framework, which was used by more than half of the schools (58.9%), followed by the FIP framework (27%).

**Table 1.** Demographic characteristics of pharmacy schools in the Eastern Mediterranean Region (N = 38).

Characteristics	N (%)
**Country (n = 36)**	
Egypt	3 (8.3)
Iraq	1 (2.8)
Islamic Republic of Iran	2 (5.6)
Jordan	4 (11.1)
Kuwait	2 (5.6)
Lebanon	2 (5.6)
Libyan Arab Jamahiriya	1 (2.8)
Pakistan	3 (8.3)
Palestine	3 (8.3)
Saudi Arabia	7 (19.4)
State of Qatar	1 (2.8)
Sudan	2 (5.6)
United Arab Emirates	4 (11.1)
Yemen	1 (2.8)
**Type of pharmacy school (n = 38)**	
Private	19 (50.0)
Public	19 (50.0)
**Academic title (n = 35)**	
Dean	9 (25.7)
Vice Dean	3 (8.6)
Director	1 (2.9)
Professor	21 (60.0)
Researcher	1 (2.9)
Offered programmes* (n = 38)	
BSc	22 (57.9)
MSc	22 (57.9)
PharmD	25 (65.8)
PhD	11 (28.9)
**Language of educational instruction (n = 37)**	
English	34 (91.9)
English/Arabic	1 (2.7)
Persian	2 (5.4)

*Participant could select multiple options.

**Table 2.** Accreditation status and competency framework adaptation in pharmacy schools in the Eastern Mediterranean Region (N = 38).

Characteristics	N (%)
**Accredited (n = 38)**	
No	7 (18.4)
Yes	31 (81.6)
**Implement CBE (n = 38)**	
No	14 (36.8)
Yes	24 (63.2)
Follow any competency framework^a^ (n = 38)	
No	16 (42.1)
Yes	22 (57.9)
**Followed framework^b^ (n = 22)**	
AACP-CAPE	7 (31.8)
AACP-CAPE and Joint Accreditation from the Ministry of Higher Education, UAE	1 (4.5)
AACP-CAPE and NCAAA	1 (4.5)
AFPC	1 (4.5)
AFPC and NAPRA	1 (4.5)
FIP and AACP-CAPE	3 (13.6)
FIP and AACP-CAPE and PPA	1 (4.5)
FIP and AFPC and NAPRA	1 (4.5)
FIP and Sudan Federal Ministry of Health Competency Framework	1 (4.5)
Iranian Ministry of Health	1 (4.5)
NAPRA	1 (4.5)
NAQAAE	3 (13.6)

Notes: AACP-CAPE = The Educational Outcomes of American Association of Colleges of Pharmacy Center for the Advancement of Pharmacy Education; CBE = Competency-Based Education, FIP = International Pharmaceutical Federation; NAPRA = National Association of Pharmacy Regulatory Authorities; NAQAAE = National Authority for Quality Assurance; NCAAA = National Center for Academic Accreditation and Evaluation; PPA = Planning, Preparation and Assessment; UAE = United Arab Emirates

^a^ Schools that do not implement CBE could not complete the survey.

^b^ Schools that implement CBE could answer this question.

#### FIP GbCF domains incorporation in pharmacy curricula across the EMR

The incorporation of the four domains of the FIP GbCF was analysed for all 24 pharmacy schools that reported implementing CBE in their curricula (Table 3). For domain 1, Pharmaceutical Public Health, 91% of the schools reported high incorporation of ‘medicines information and advice' competency, followed by ‘health promotion' competency (87.5%). However, 58.3% reported minimal incorporation of the ‘emergency response' competency. Under domains 2 and 4, all the sub domains were highly incorporated, with incorporation rates ranging between 79% and 95.8%. Whereas in domain 3 (Organisation and Management), the majority of competencies were highly incorporated, yet 25% of schools reported minimal incorporation of some sub domains, such as ‘budget and reimbursement' and ‘workplace management'.

**Table 3.** Level of incorporation of FIP Global Competency Framework into the pharmacy programmes curriculum that implement competency-based education in the Eastern Mediterranean Region (N = 24).

			
Competency	Total responses (N)	Minimally incorporated (0–2)	Highly incorporated (3–5)
		N (%)	N (%)
**Domain 1: Pharmaceutical Public Health**			
Emergency response:	24	14 (58.3)	10 (41.6)
Health promotion:	24	3 (12.5)	21 (87.5)
Medicines information and advice	24	2 (8.3)	22 (91.6)
**Domain 2: Pharmaceutical Care**			
Assessment of medicines	24	4 (16.6)	20 (83.3)
Compounding medicines	24	3 (12.5)	21 (87.5)
Dispensing	24	2 (8.3)	22 (91.7)
Medicines	24	1 (4.2)	23 (95.8)
Monitor medicines therapy	24	3 (12.5)	21 (87.5)
Patient consultation and diagnosis	24	3 (12.5)	21 (87.5)
**Domain 3: Organisation and Management**			
Budget and reimbursement	24	6 (25)	18 (75)
Human resources management	24	5 (20.8)	19 (79.2)
Improvement of service	24	4 (16.7)	20 (83.3)
Procurement	24	3 (12.5)	21 (87.5)
Supply chain management	24	5 (20.8)	19 (79.2)
Workplace management	24	6 (25)	18 (75)
**Domain 4: Professional/Personal**			
Communication skills	24	1 (4.2)	23 (95.8)
Continuing Professional Development (CPD)	24	3 (12.5)	21 (87.5)
Digital literacy	24	5 (20.8)	19 (79.2)
Inter-professional collaboration	24	2 (8.3)	22 (91.7)
Leadership and self-regulation	24	2 (8.3)	22 (91.7)
Legal and regulatory practice	24	4 (16.7)	20 (83.3)
Professional and ethical practice	24	1 (4.2)	23 (95.8)
Quality assurance item	24	4 (16.7)	20 (83.3)

#### Perceptions of facilitators and barriers

Table 4 summarises the perception of facilitators and barriers to implementing CBE. Almost all facilitator items were highly rated as facilitators by the majority of the schools, indicating strong support for CBE implementation. Conversely, the perception of barriers to CBE implementation varied between the schools. The highest perceived barrier among participants was the need for sufficient manpower resources (77.3%), followed by the requirements for significant resource allocation for training (72.7%). The least perceived barrier was the concern that students may be inclined to achieve only the minimum competency requirements (40.9%).

**Table 4.** Integration of qualitative and quantitative findings.

Table 4.1 Facilitators and perceived facilitators for CBE implementation				
Item	Quantitative phase	Qualitative phase		
Level of resources dedicated to CBE implementation and ongoing operations, such as funding, training, education, physical space, and time	Minimally perceived as a facilitator vs. highly perceived as a facilitator	Theme	Category	Quote
	4.5% vs 95.5%	F2: Resources and training opportunities	Fund Physical space Training and education	‘The university administration provides us with financial support which is upgraded yearly’ (CBE 6) ‘We believe that the state-of-the-art facility we have at our campus,which is a smart campus … .supports the implementation of CBE’ (CBE 4) ‘Faculty training through pharmacy education workshops really helps support them towards CBE implementation'. (non-CBE 1)
Availability of necessary manpower, including administers, faculty, and stakeholders, to develop, implement, evaluate the curriculum and provide proper training	4.5% vs 95.5%	F1: Leadership and vision F3: Student and faculty experience F4: Communication, collaboration, and engagement	Administers Faculty Stakeholders Proper training	‘Our Dean is always on board with new advancement in teaching and education. He gives us the space to explore new approaches and makes sure we have the support we need' (CBE 4) ‘You need faculty who really understand what CBE is all about. It’s not just about changing the syllabus—it’s a whole different mindset, and having experienced people makes a big difference.' (non-CBE 3) ‘Working with people from other universities really helps, whether it's course reviewers, guest lecturers, or joint student activities. This kind of collaboration helps align our program with broader standards and expectations.' (non-CBE 4) ‘Faculty need opportunities to attend pharmacy education workshops for their learning and development’. (non-CBE 8)
Local practice-related polices supporting the need for competent pharmacy graduates	9.1% vs 90.9%	F2: Resources and training opportunities	National organisation	‘[Local] bodies didn’t directly make the change but their guidelines and standards provide us supportive framework that made it easier to map our CBE implementation with national expectations.' (CBE 8)
Local education-related policies supporting CBE in pharmacy	9.1% vs 90.9%	F3: Student and faculty experience	Teaching methodologies	‘Teaching and learning methods and evaluation strategies like student projects, peer reviews, and multiple forms of assessment, really help in implementing CBE. These methods are encouraged by our local education-related policies, so they give us a strong foundation to build our CBE.' (CBE 4)
Community support and appreciation of the emerging pharmacists’ role	18.2% vs 81.8%	None		
External stakeholders support students’ educational and training experience	13.6% vs 86.4%	F4: Communication, collaboration, and engagement	Local support National support International support	‘External reviewers from other local universities for our courses and exams, help in teaching process and curriculum delivery, shared students’ activities that address some of our competencies' (CBE 8) ‘Working with the country’s pharmaceutical association has been super helpful. They back us up, keep us in line with what’s needed nationally, that really boost our CBE work' (CBE 6) ‘We have good connections with other universities in the region. I believe these relationships will really help us share ideas, learn from their experiences, and get support as we work on implementing CBE.' (non-CBE 5)
Students’ perception towards advancing their educational training	13.6% vs 86.4%	F3: Student and faculty experience	Students experience Students awareness	‘From what we’ve seen, students really enjoy and appreciate CBE because it connects what they learn directly to real-life practice. They feel more confident when they understand how their studies apply outside their classroom' (CBE 2) ‘Our students are aware that education is changing. They know that traditional methods are not enough anymore and seem ready to have new approaches like competency-based CBE' (non-CBE 8)
Incentives such as rewards, and promotions for individuals involved in CBE development and implementation	22.7% vs 77.3%	None		
Commitment of individuals involved in CBE development and implementation	4.5% vs 95.5%	F1: Leadership and vision F3: Student and faculty experience	Leadership Faculty Accreditation and quality assurance organisation	‘The Dean and Vice Deans play a huge role in making CBE happen—they provide support, and make sure everyone stays focused on the goals. Without their guidance, it would be much harder to get things moving.' (non-CBE 5) ‘If they [faculty] don’t really believe in the value of CBE and stay committed, it will be hard to make a change.' (non-CBE 7) ‘The national accreditation and quality assurance organization is very organized, dedicated, supportive, and have very good training center, support all our needs’. (CBE 7).
Individuals’ belief in their own capabilities	4.5% vs 95.5%	None		
Norms and values of the institution supporting CBE in pharmacy	9.1% vs 90.9%	F1: Leadership and vision	Culture	The culture of our institution supports innovation and continuous improvement, which makes it easier to implement CBE because everyone shares that vision for advancing pharmacy education (CBE 1)
Competitive pressure to implement CBE (i.e. other key peer or competing pharmacy schools have already implemented it)	13.6% vs 86.4%	None		

Table 4.2 Barriers and perceived barriers for CBE implementation				
Item	Quantitative Phase	Qualitative Phase		
Requires delineation and operationalisation of competencies that can be difficult to define, develop, implement, and assess	Minimally perceived as a barrier vs highly perceived as a barrier	Theme	Category	Quote
	40.9% vs 59.1%	B3: Demanding and time and workload challenges B4: Lack of vision clarity and planning	Competency mapping Competency assessment	‘Mapping the competencies wasn’t straightforward – it took a lot of back and forth to figure out where each one fits within the courses. It required a full review of learning outcomes, assessments, and even teaching methods' (CBE 2) ‘I think assessing competencies is hard task. It takes a lot of time and effort to design tools that actually measure skills, not just knowledge. It’s a whole different approach from traditional exams.' (non-CBE 5)
Extensive change of traditional curricula to a new model that necessities the application of new teaching methods and assessment strategies	40.9% vs 59.1%	B3: Demanding and time and workload challenges		‘Moving from the traditional curriculum to CBE was not just a small adjustment. We had to restructure assessments, rubrics, evaluation criteria’ and make new assessments and teaching methods' (CBE 7)
Time-consuming (e.g. time constraints that may interfere with faculty providing adequate support to students)	40.9% vs 59.1%	B3: Demanding and time and workload challenges		‘Implementing CBE takes a lot more time than we expected. It involves many tasks from curriculum redesign to training faculty and developing new assessments. It’s not something you can rush.' (CBE 8)
Necessitates sufficient manpower resources such as administrators, faculty, and stakeholders	22.7% vs 77.3%	B1: limited resources	Administrators Faculty	‘We are expected to implement CBE, but university and college administration are not supportive’. No extra staff, no training. How are we supposed to succeed without support and guidance?' (non-CBE 3) ‘You cannot do CBE properly without enough people to make it happen. You need teachers who know how to teach it, staff to organize everything, and leaders who actually support it.' (non-CBE 7)
Requires significant dedication of resources and funding to meet the requirements to develop, implement, and evaluate the curriculum	31.8% vs 68.2%	B1: limited resources	Fund Physical space	‘We fully believe in the value of CBE and we have the plans, however, with limited budget things might be difficult to implement. This is issue is beyond our control.' (non-CBE 1) ‘Our classrooms are note built for this kind of learning. CBE needs flexible spaces where students can collaborate, move around, and work on real tasks – but we’re stuck in old lecture halls with fixed chairs. Even simple things like grouping tables or setting up workstations become a struggle (non-CBE 3)
Requires significant dedication of resources to provide the proper training (e.g. new faculty may need to be hired and instructors will require faculty development related to CBE)	27.3% vs 72.7%	B1: limited resources	Training sitesF aculty development	‘Hospitals that students should be trained at should be accredited by the Ministry of Health, students can’t be trained without at non-accredited hospital even if it is a top hospital in the country' (CBE 6) ‘Faculty should be educated and trained about CBE … The information about CBE is there, and we are applying it, but faculty might not really understand it, taking into consideration the faculty with pharmaceutical science background' (CBE 3)
Students may be inclined to reach only the minimum requirements for the defined competencies	59.1% vs 40.9%	B2: Resistance to change		‘Current generation of pharmacy students seems more focused on passing exams and completing their program rather than obtaining the skills and knowledge required by the modern healthcare systems’. (CBE 4)

### Qualitative phase

In the qualitative phase, interviews were conducted with 18 participants from nine different countries who implemented and those who did not implement CBE. Participants varied in their country of work, gender, age, and type of pharmacy school (private vs. public), providing a range of perspectives relevant to the study objectives. The participants held various academic leadership positions, including Deans and Vice Deans. The interviews focused on two main domains: facilitators and barriers to CBE implementation.

For the domain of ‘facilitators (F)', four themes emerged from schools implementing and those not implementing CBE.

***Theme F1 ‘Leadership and vision*’:** Leadership was recognised as a key facilitator. Deans were specifically mentioned by several participants as critical drivers of CBE implementation due to their influential role in advancing educational reforms and setting institutional vision. As one participant noted: ‘Some people in leadership positions, like the Dean or counselor, are key pillars towards advancing the education. Their support and commitment can significantly influence the successful implementation of initiatives like CBE’ (CBE 3). Other interviewees identified the attitude of Deans in facilitating CBE implementation, as one noted: ‘The Dean and Associate Dean were really supportive. They encouraged us from the beginning and provided clear strategic guidance on how to move forward with CBE (CBE 8)'. The importance of school vision and its translation to hiring staff with CBE experience were also noted: ‘Having a clear vision for the college and leadership that focuses on hiring staff with CBE experience really makes a big difference in implementing CBE (non-CBE 1)'*.* The preparedness of colleges was noted as a facilitating factor, especially in relation to presenting the case for university administration ‘The college should be well prepared to make it easier to present a strong case to the university administration about the need for CBE' (non-CBE 5). Another participant added that having clear guidance and a plan can also support CBE implementation: ‘The college already had a well-defined CBE implementation plan, so we knew exactly what steps to take and how to get started' (CBE 1).

***Theme F2 ‘Resources and training opportunities'***: There was a clear indication that resources and staff and faculty training play a key role in the adoption of CBE. Participants highlighted different types of supports such as sustained funding, described as ‘The university administration provides us with financial support which is upgraded yearly' (CBE 6) and ‘All these new technologies and improved teaching facilities like simulation labs requires proper funding' (non-CBE 3)*.* Other sources of support, such as the accreditation bodies themselves, were recognised as a major external resource that was necessary to support the implementation of CBE, with one participant noting: ‘The national accreditation and quality assurance organization is very organized, dedicated, supportive, and have very good training centre, support all our needs' (CBE 7). Participants also mentioned facilities while noting the investment of innovation in the mentioned facilities that helped set the stage for CBE adoption and implementation: ‘We believe that the state-of-the art facility we have at our campus which is a smart campus: simulation programs, simulation labs mainly in clinical pharmacy field, drug design programs, learning management system model, and AI supports the implementation of CBE’ (CBE4). Faculty and staff training was evident as an enabler for CBE implementation in the responses from participants to the question about facilitators as one participant, for instance, mentioned: ‘Faculty members need opportunities to attend pharmacy education workshops for their learning and development' (non-CBE 8). Participants also mentioned the awareness about advancements in pharmacy education that warrant the adoption and implementation of CBE as encapsulated it: ‘It’s about identifying the services our graduates need to provide … and being aware of how education and practice are advancing' (CBE 2). The availability of experienced faculty in CBE was recognised as a potential facilitator ‘Maybe it would be good to have more professors who have actually worked with this kind of curriculum before. When faculty know how CBE works in practice, it's much easier to get things moving in the right direction' (non-CBE 2). Exposure to institutions that have successfully implemented CBE was noted as a facilitator to learn from best practices, as one of the interviewees stated: ‘It is always good to get exposure and experience from the people who have already implemented it. Seeing how others did it, the steps they took, the challenges they faced, gives us a clearer picture and helps us avoid starting from scratch’ (CBE 5).

***Theme F3 ‘Student and faculty experience':*** The positive experience noted from examples related to students and faculty supported CBE implementation. For students, the assessment, revised curriculum, and focus on skills and a practice-laden approach created a more engaging and relevant experience. This was best captured by two responses from different participants: ‘Students liked the assessments as they are flexible, enjoyable, and real-life ones. That made the learning feel more meaningful to them' (CBE 1) and ‘Students don’t cause a challenge if things are planned properly and balanced between lectures, practice, simulation or practical labs. When they see the purpose behind it, they actually respond really well' (CBE 7). In addition, the progressive mindset of students was evident as one facilitator for potential CBE adoption. Participants described students as being aware of the advanced healthcare system and its demands, including bridging the gap between academia and real-world practice. This awareness was reflected in students’ attitudes toward the change, as one participant noted: ‘Our students speak a lot about the disconnect between academic life and the real-world pharmaceutical practice. They’re aware of it and want something more aligned with what they’ll actually face out there' (non-CBE 6). Students were also perceived as open and eager for change, particularly in relation to CBE, as highlighted by one of the interviewees: ‘I believe our students like might be somehow interested in such a change. They’re ready for something more practical and meaningful' (CBE 2). Faculty also cooperated with the change towards CBE due to vision clarity and organized planning: ‘No resistance [from faculty] as college involved the members in the early planning stage, and made sure everyone understood the vision and what the change to CBE would mean' (CBE 3). Faculty background about accrediting bodies was highlighted as another enabler, as one participant noted: ‘our expectations [faculty] from international accrediting bodies would be to support our program by all means … ' (non-CBE 4).

***Theme F4*** ***‘Communication, collaboration and engagement'*****:** Communication, collaboration, and engagement were identified as key facilitators for implementing CBE. Collaboration with other entities, particularly external stakeholders, was mentioned by a number of participants. The stakeholders included, reviewers from local universities ‘External reviewers from other local universities for our courses and exams, help in teaching process and curriculum delivery, shared students’ activities that address some of our competencies' (CBE 8) and connection to other pharmacy schools with experience in CBE ‘College connected with pharmacy schools internationally (America and Europe) with experience in CBE. This connection has been really valuable because we get to see how they have addressed challenges, what worked for them, and get useful ideas to improve our own program. It’s like having mentors' (CBE 1). Collaboration with accreditation bodies was noted by participants to be an opportunity for guidance and structured support: ‘Our experience with accreditation bodies suggests they would be supportive’, and ‘Our relationship with the accrediting body would be pivotal' (non-CBE 4). Furthermore, alliance with regional and international organizations was noted as a facilitating factor: ‘We just connected with some experts from outside from the Pharmacy International Federation. That connection is really helpful in guiding and supporting our CBE effort' (non-CBE 8).

The second domain ‘barriers (B)' revealed five themes that emerged from schools implementing and those not implementing CBE.

***Theme B1*** *‘**Limited resources'*****:** The availability and suitability of physical spaces and facilities were identified as key barriers for CBE implementation. Participants mentioned a shortage in essential infrastructure, particularly in areas such as teaching laboratories, learning spaces, and simulation facilities, as one participant noted: ‘teaching facilities like teaching labs, spaces, simulation (are lacking) which makes it harder to deliver hands-on, competency-based training the way it is meant to be' (non-CBE 6). Another noted barrier was the financial constraints. One participant noted: ‘the university is running a chronic financial problem, we don't have any support from the government’. Another participant also highlighted: ‘We are working to implement CBE with a limited budget. This means that we have to prioritize certain aspects first and work on other aspects gradually as funding becomes available' (non-CBE *4).* A set of other human resource-related barriers was evident. For example, staff number shortage due to ongoing country conflicts made it hard to meet accreditation requirements ‘Low number of staff due to country conflict was difficult for [accrediting] bodies to understand which made it really challenging for us to meet their standards and obtain the accreditation on time' (CBE 1). Additionally, hospital readiness to train students as part of CBE requirements was problematic, as one participant clarified: ‘Hospitals that students should be trained at should be accredited by the Ministry of Health, students can’t be trained without at non-accredited hospital even if it is a top hospital in the country’ (CBE 6).

***Theme B2 ‘Resistance to change':*** Resistance to change in different forms was mentioned as a barrier to implementing CBE. For example, pharmacy associations and their vision were noted as a form of resistance to change by one participant: ‘We had not much support from the pharmacy associations could be due to different visions. While we were pushing for modern education, they seemed stuck in traditional models. There was no guidance on assessments, no advocacy for policy changes' (non-CBE 6). Yet this resistance dissipated over time as further clarified by the same participant: ‘There is resistance but it is decreasing with time' (non-CBE 6). Another significant barrier to CBE implementation, as highlighted by participants, was faculty resistance. Shifting the faculty mindset away from traditional teaching approaches was seen as a challenge, with one interviewee stating: ‘It's hard to move people from their conventional mindset, some faculty see CBE not as progress but as criticism of how they've always done things’ (non-CBE 1). It was further acknowledged that faculty resistance to change might stem from a perceived threat to their professional identity, as mentioned by one of the interviewees: ‘Many faculty members would likely view this sudden change as a threat to their professional identity' (CBE 7). Students mindset with regards to academic survival was one of the highlighted barriers, as described by one participant: ‘Current generation of pharmacy students seems more focused on passing exams and completing their program rather than obtaining the skills and knowledge required by the modern healthcare systems' (CBE 4). Furthermore, the traditional education was noted to be a passive barrier for educational innovation, as described by one interviewee: ‘Students are used to this conventional system since their school. This might affect their willingness to accept new educational system like CBE' (non-CBE 2).

***Theme B3: ‘Demanding and time and workload challenges':*** Participants mentioned that the changes associated with CBE implementation are heavy and posed challenges for leadership, as one participant mentioned: ‘Leadership [head of departments] faced many issues in terms of resources, faculty workload, administrative work that came with CBE implementation' (CBE 6). Faculty members were also affected, particularly in relation to mapping the current course assessments to the competencies, as one participant stated ‘Mapping the assessments with each competency was really time-consuming. It wasn’t just about adjusting exams – it required rethinking how we evaluate learning to make sure it matches the required competencies' (CBE 3). The demanding schedule of faculty members was noted to be a challenge to dedicate time to developing and implementing CBE, as one participated clarified: ‘faculty being overwhelm is a real concern. With teaching, research, and administrative duties, it is really hard to find time for redesigning courses and creating new assessments that align with CBE’ (non-CBE 5). Further, participants found challenges in the need to make many changes to fulfil CBE implementation requirements, including changes in evaluation and teaching methods, as one participant elaborated, We had to create entirely new assessments and rethink our teaching methods to match the CBE approach. It’s a big shift from what we were used to’ (CBE 3).

***Theme B4 ‘Lack of vision clarity and planning':*** Lack of clarity at different levels was identified as a barrier to the implementation of CBE. One interviewee stated that: ‘Vision was not clear initially, which made the faculty and administrators struggle to understand the goals of the transition to CBE' (CBE 1). This ambiguity was also on the level of how the change was justified, as one participant emphasized: ‘Communicating the significance of this change was a challenge. People understood the plan but not its importance' (CBE 2). Similarly, delays and time pressure were noted as barriers to CBE implementation, as clarified by one interviewee: ‘While [college administration was] supportive, we had issues with practical assistance – sometimes there was a delay or unclear plan’ (CBE 7). ‘These resulted in late communication of CBE changes to students, as further clarified by the participant: ‘Students received the instructions of the implementation just 1-2 months ahead.' (CBE 7).

***Theme B5 ‘Political turmoil':*** Some participants noted the impact of political instability on the implementation of CBE, as one interviewee highlighted: ‘Ongoing Conflicts [in the country] makes it very difficult to focus on educational improvements like implementing CBE as resources are directed to towards safety concerns'(non-CBE 8) and ‘Given the country’s situation – we are living under war – it’s really challenging to prioritize and successfully implement educational changes like CBE' (non-CBE 3). This political factor was acknowledged to preoccupy the university administration, leaving little room for educational reforms as mentioned by one participant: ‘University administration are dealing with bigger issues than educational models. We're talking about keeping the lights on, ensuring faculty can get to campus safely' (non-CBE 8). Another interviewee highlighted that the economic instability during political turmoil was another barrier: ‘managing resources during economic instability is a big challenge. With constant budget cut and uncertainty, it is hard to plan ahead for something comprehensive like CBE' (non-CBE 3). In addition, one participant pointed to the difficulty of competency mapping and national coordination due to governmental conflicts:

Competency mapping was really difficult because of the governmental conflicts we had for years. There were also challenges in applying anything at the national level unless you had an official embassy involved. One major issue was the lack of a National Academic Reference Standard (NARS), which was missing due to the instability. But despite all that, the college managed to succeed in developing its own NARS because it was well-prepared and committed to moving forward. (CBE 1)

The quantitative and qualitative data integration is illustrated in Table 4.

## Discussion

CBE is an evolving model in health professions education, including pharmacy, that shifts the focus from time-bound learning to the achievement of clearly defined competencies. In essence, the primary goal of CBE is to ensure that graduates possess the necessary skills, knowledge, and attitudes required for real-world practice (L. D. Gruppen et al., 2016; Katoue & Schwinghammer, 2020; McMullen et al., 2023). In this study, greater interpretive emphasis was placed on the qualitative findings, which provided a deeper contextual understanding of the quantitative results. The study found that the implementation of CBE is prevalent across the EMR, with pharmacy schools reporting successful integration of most domains outlined in the FIP GbCF. Deficiencies were identified, however, in areas such as emergency response and selected management-related competencies. Furthermore, the integrated data in the current study indicated that while there are several facilitators promoting CBE adoption, substantial barriers continue to challenge its full implementation and sustainability. The EMR encompasses countries with markedly diverse health systems and economic classifications, ranging from high-income countries with advanced regulatory and healthcare infrastructures to lower-income and conflict-affected countries with fragmented systems and limited resources (Obaid et al., 2022; World Health Organization, 2020). This diversity also extends to the regulation of pharmacy practice and polices, where in some countries prescription-only medications are dispensed without a prescription (Bajis et al., 2016, 2018; Hussain et al., 2025). Such heterogeneity directly influences the roles, expectations, and competencies required of pharmacists across the region. In this context, CBE provides a structured, outcomes-oriented approach that can help standardise pharmacist preparation while allowing for contextual adaptation to national health priorities (Katoue & Schwinghammer, 2020). By focusing on fundamental competencies such as patient counselling, health promotion, and ethical practice, CBE ensures that pharmacy graduates are equipped to contribute effectively to diverse healthcare delivery models and respond to public health challenges (L. D. Gruppen et al., 2016). Therefore, this study is particularly useful as it offers region-wide evidence on current CBE implementation, identifies systemic gaps, and highlights opportunities for aligning pharmacy education with the health system needs of EMR countries.

As highlighted in this study, effective leadership plays a pivotal role in driving educational transformation, particularly in implementing innovative models like CBE. Academic leaders, such as deans, play a central role in setting the vision and strategic directions necessary for CBE adoption. This is consistent with a previous study indicating that academic leadership is one of the key factors for the successful implementation and sustained use of CBE, due to the leaders’ clear understanding of the necessary disruption required for meaningful change (Bray et al., 2017). According to Halimah et al., effective academic leaders are not only visionaries but also catalysts for innovation, fostering adaptability, creativity, and collaboration throughout the transformation process (Halimah et al., 2024).

The availability of various resources such as funding, technologies, innovative teaching strategies, including utilisation of artificial intelligence (AI), and accrediting bodies was identified as one of the important facilitators of implementing CBE. Although not frequently emphasized by the participants, the integration of AI was highlighted as one of the enablers for CBE implementation. AI holds a transformative potential in CBE by enabling personalised and adaptive learning experiences (Narayanan et al., 2023). While AI has shown great promise in CBE, more rigorous research is needed to identify the most effective AI tools in the context of medical and health professions education (Tozsin et al., 2024; Varma et al., 2023). It is worth noting that digital literacy, which confines the ability to utilise and interact with digital technology, including AI (Uyu et al., 2024), is one of the core competencies incorporated in the FIP GbCF, signifying its relevance in preparing competent and capable future healthcare professionals (International Pharmaceutical Federation, 2023).

Similarly, patient simulation was recognised as a facilitator for CBE application, although it was not commonly mentioned by our participants. Simulation-based education has increasingly been recognised as a cornerstone to support that shift toward CBE models. Studies have found that simulation serves as a highly effective tool in not only improving educational outcomes but also addresing various logistical challenges in contemporary medical education (Chacko, 2017; Satava, 2009). The role of simulation in CBE was further supported during and after the COVID-19 pandemic, as its use became pivotal due to a marked decrease in student–patient interaction (Kapoor et al., 2021). One of the most frequently cited facilitators of CBE implementation in this study was the support provided by the accreditation bodies. The published literature confirms this finding and supports the significance of accreditation systems in enabling the transition to CBE. It is found that key design elements such as alignment with educational theory, emphasis on quality assurance and continuous improvement, and structured evaluation processes offer valuable opportunities to advance this shift to CBE adoption (Bandiera et al., 2020; Dalseg et al., 2024; Frank et al., 2020).

From the viewpoint of pharmacy schools that participated in this study, the progressive mindset of their students was seen to be an enabler for potential CBE adoption. The perception of pharmacy students towards CBE is evident from the existing literature (Al-Haqan et al., 2021; Allen et al., 2016; Bray et al., 2017; Kary et al., 2019; McMullen et al., 2023; Ortega-Dela Cruz, 2022). For example, a systematic review that examined CBE-related approaches worldwide found that students valued various aspects of CBE, including its flexibility and learner-centered approach, practical assessments, and the immediate and visual feedback using advanced tools (McMullen et al., 2023). Similarly, in another study that explored undergraduate and postgraduate students’ attitudes towards CBE, it was found that more than half of the participants expressed favourable views towards CBE, believing that it better prepared them for the workplace, raised academic standards, and emphasized the importance of industry collaboration to bridge the gap between theory and real-world practice (Ortega-Dela Cruz, 2022).

This study revealed several challenges for CBE adaptation. Although the availability of resources was one of the main facilitators for CBE implementation, their limited availability poses a challenge for some schools. Participants in this study highlighted that the lack of facilities, funds, and human resources can hinder themeeting of the requirements of CBE implementation, including curricular design, teaching, and assessment. Furthermore, shifting faculty perspectives beyond conventional teaching was identified as a challenge. These findings are consistent with Brownell and Tanner's (2012) findings in their comprehensive analysis of barriers to pedagogical change in higher education (Brownell & Tanner, 2012). They noted that faculty resistance and resource limitations consistently undermine educational reform efforts regardless of institutional readiness, highlighting the need for targeted interventions that specifically address these persistent obstacles. Faculty resistance to shift from conventional teaching mindsets, potentially viewing the transition as a threat to their professional identity, was also supported in the literature (Curry & Docherty, 2017; Gruppen et al., 2016).

Another highlighted barrier in this study was the workload and time-allocation associated with CBE implementation. This barrier was emphasized by Medina (2017), who noted that CBE may strain faculty resources, making it challenging to balance assessment demands with teaching responsibilities and other academic duties (Medina, 2017).

Participants also highlighted how political turmoil stands as a particularly disruptive barrier towards CBE implementation. This finding is supported by previous research, which has shown that political instability and economic challenges in the EMR can hinder the advancement of educational initiatives. In such contexts, university administrations are often preoccupied with maintaining basic operations, leaving them with limited capacity to implement curriculum reforms (Bajis et al., 2018).

This study has several strengths. First, the use of a mixed-methods approach allowed the researchers to integrate both quantitative and qualitative data, offering a more comprehensive understanding of participants' perspectivs (Wasti et al., 2022). Second, the use of a self-administered questionnaire has the advantage of being easily administered to a large number of participants within a short time period, and helps eliminate interviewer and social desirability bias (Kuphanga, 2024). In addition, this technique is effective in capturing participants’ behaviours and perceptions, particularly in educational research settings. Third, although the response rate was low, including all the pharmacy schools in the EMR helped in reflecting the diversity of educational systems, institutional capacities, and national contexts across the EMR. It further helps in the identification of the common barriers and facilitators to CBE implementation. However, the findings of this study should be interpreted with caution due to several limitations. The quantitative part of the study had a low response rate, which could affect the generalizability of the results. In addition, the quantitative component involved a relatively small number of responding pharmacy schools, which may have limited statistical power and the ability to draw strong inferential conclusions. Therefore, the quantitative findings should be interpreted with caution and considered complementary to the qualitative insights rather than standalone evidence. Additionally, the unequal participation across EMR countries, with some countries absent and others contributing multiple schools (e.g. Saudi Arabia and the United Arab Emirates), may limit the regional generalizability of the findings. It is worthwhile to note that at the time of the study, a few participants had recently been appointed to their positions or had just joined the institution, which limited their ability to provide a comprehensive perception of the CBE status at their institution. Another limitation of this study is that only schools that implemented CBE at the time of the study completed the online survey. This may limit direct comparison with schools that didn’t implement CBE. Nonetheless, non-CBE schools were included in the qualitative phase, providing valuable insights into perceived barriers and readiness factors that complemented the quantitative findings. In addition, the time gap between the quantitative and qualitative phases may have introduced recall bias or reflected contextual changes in educational policies, accreditation, or curricula during that period.

## Conclusion

CBE in pharmacy schools focuses on developing students’ skills, knowledge, and professional attitudes to meet healthcare needs, with an emphasis on outcomes rather than time-bound progression. Key enablers of effective CBE implementation include well-defined competency frameworks, institutional leadership support, active stakeholder engagement and collaboration, faculty and student experience, and availability of various resources, including faculty development. Schools considering the adoption of CBE should provide strong justifications, secure financial and administrative support, and address faculty concerns through targeted training and exposure to successful models. Overcoming these barriers will require strong institutional leadership, faculty commitment, and a strategic and evidence-based approach to ensure a smooth and sustainable transition to effective CBE.

## Data Availability

The authors confirm that the data supporting the findings of this study are available within the article.
